# Post-mortem study of the association between cardiac iron and fibrosis in transfusion dependent anaemia

**DOI:** 10.1186/s12968-017-0349-3

**Published:** 2017-03-27

**Authors:** Paul Kirk, Mary Sheppard, John-Paul Carpenter, Lisa Anderson, Taigang He, Tim St Pierre, Renzo Galanello, Gualtiero Catani, John Wood, Suthat Fucharoen, John B Porter, J Malcolm Walker, Gian Luca Forni, Dudley J Pennell

**Affiliations:** 1 Cardiovascular Magnetic Resonance Unit, Royal Brompton Hospital, Sydney Street, SW3 6NP, London, UK; 2 National Heart and Lung Institute, Imperial College, London, UK; 3 CRY Centre for Cardiac Pathology, Royal Brompton Hospital, London, UK; 4 University of Western Australia, Perth, Australia; 5 Ospedale Microcitemico, Cagliari, Italy; 6 Children’s Hospital, Los Angeles, USA; 7 Mahidol University, Bangkok, Thailand; 8 The Hatter Cardiovascular Institute, University College Hospital, London, UK; 9 Centro della Microcitemia, Ospedale Galliera, Genoa, Italy

**Keywords:** Thalassaemia, Cardiac siderosis, Cardiac MR, Iron, Heart, Fibrosis, Histopathology

## Abstract

**Background:**

Heart failure related to cardiac siderosis remains a major cause of death in transfusion dependent anaemias. Replacement fibrosis has been reported as causative of heart failure in siderotic cardiomyopathy in historical reports, but these findings do not accord with the reversible nature of siderotic heart failure achievable with intensive iron chelation.

**Methods:**

Ten whole human hearts (9 beta-thalassemia major, 1 sideroblastic anaemia) were examined for iron loading and fibrosis (replacement and interstitial). Five had died from heart failure, 4 had cardiac transplantation for heart failure, and 1 had no heart failure (death from a stroke). Heart samples iron content was measured using atomic emission spectroscopy. Interstitial fibrosis was quantified by computer using picrosirius red (PSR) staining and expressed as collagen volume fraction (CVF) with normal value for left ventricle <3%.

**Results:**

The 9 hearts affected by heart failure had severe iron loading with very low T2* of 5.0 ± 2.0 ms (iron concentration 8.5 ± 7.0 mg/g dw) and diffuse granular myocardial iron deposition. In none of the 10 hearts was significant macroscopic replacement fibrosis present. In only 2 hearts was interstitial fibrosis present, but with low CVF: in one patient with no cardiac siderosis (death by stroke, CVF 5.9%) and in a heart failure patient (CVF 2%). In the remaining 8 patients, no interstitial fibrosis was seen despite all having severe cardiac siderosis and heart failure (CVF 1.86% ±0.87%).

**Conclusion:**

Replacement cardiac fibrosis was not seen in the 9 post-mortem hearts from patients with severe cardiac siderosis and heart failure leading to death or transplantation, which contrasts markedly to historical reports. Minor interstitial fibrosis was also unusual and very limited in extent. These findings accord with the potential for reversibility of heart failure seen in iron overload cardiomyopathy.

**Trial registration:**

ClinicalTrials.gov Identifier: NCT00520559

## Background

The transfusion dependent anaemias cause a substantial burden of morbidity and mortality, of which thalassaemia is the commonest single gene disorder worldwide [1]. The transfusions that are necessary for survival cause tissue iron loading, and this ultimately results in heart failure as the major cause of death [2, 3]. Histopathological studies of patients with cardiac siderosis have indicated that replacement myocardial fibrosis is prominent and causative of the heart failure [4, 5], but these data were based on patients who died 50 years ago or more and prior to the modern era of iron chelation treatment. These historical data have now become controversial because it is now recognised that the risk of developing heart failure in beta-thalassaemia major is closely associated with myocardial siderosis [6], heart failure may be fully reversed with aggressive iron-chelation treatment [7], and improvement in ventricular function occurs in concert with myocardial iron reduction implying a causal relationship [8–10]. In other non-transfusional cardiomyopathies such as hypertrophic cardiomyopathy [11], dilated cardiomyopathy [12], and arrhythmogenic right/left ventricular cardiomyopathy [13, 14], macroscopic replacement cardiac fibrosis is a prominent feature and is associated with deteriorating cardiac function and an adverse outcome [15–18]. However, improvement in left ventricular function with treatment is limited or not possible in these cardiomyopathies, which may relate to the underlying myopathy, but also because the replacement fibrosis appears to be permanent. Therefore, we hypothesized that the modern clinical finding of reversible ventricular dysfunction in myocardial siderosis is not driven by replacement fibrosis. To investigate this, we analysed hearts from transfusion dependent patients to examine for cardiac fibrosis by histology and relate the findings to iron overload cardiomyopathy.

## Methods

### Study hearts

Ten hearts were studied from 5 centres, with 9 from patients with beta thalassaemia major and 1 from a patient with sideroblastic anaemia. A separate report associating myocardial T2* to myocardial iron in these hearts has been published [19]. Six of the hearts were obtained post-mortem and 4 post cardiac transplantation. Three patients had died from heart failure, 4 had cardiac transplantation for heart failure, 2 had current heart failure but had died from other causes (1 tamponade, 1 encephalitis), and 1 had died from a stroke (no heart failure). The mean blood units transfused was 28.2 ± 9.7 per year per patient. Eight patients had received chelation therapy with deferoxamine, and 2 patients had received combination treatment with deferoxamine with deferiprone. Detailed patient demographics are shown in Table 1.

**Table 1:** Patient Demographics

Heart	1	2	3	4	5	6	7	8	9	10
Race	White	White	White	White	White	White	White	White	White	Asian
Origin	Italy	France	Italy	Portugal	Italy	Italy	Italy	Italy	Italy	Thailand
Sex	Male	Female	Male	Female	Female	Male	Male	Male	Female	Male
Death/Transplant Year	Death 2004	Death 2005	Death 1964	Death 1972	Death 1990	Tx 1995	Tx 1997	Tx 1999	Tx 2000	Death 2005
Cause of death/Transplant	Stroke	Heart failure	Heart failure	Heart failure	Heart failure	Heart failure	Heart failure	Heart failure	Heart failure	Heart failure
LV iron by spectrometry (mg/g dw)	0.38 ± 0.13	8.2 ±1.4	9.5 ±1.9	25.9 ±10.3	7.7 ±1.5	3.6 ±0.8	3.4 ±0.5	5.9 ±1.0	8.8 ±1.9	5.6 ±1.4
LV T2* (ms)	44.4 ± 5.3	4.7 ±0.6	3.7 ±0.6	2.0 ±0.4	3.6 ±0.5	8.0 ±1.0	7.7 ±1.2	4.4 ±0.6	3.9 ±0.6	5.8 ±1.4
Age at death/transplant (years old)	46	62	10	15	20	23	24	21	31	24
Diagnosis	TM	SA	TM	TM	TM	TM	TM	TM	TM	TM
Age at diagnosis (years)	2	32	4	0.25	0	1	0	0	5	2
Height (cm)	173	160	140	140	156	170	164	157	151	167
Weight (kg)	63	51	33	30	51	54	61	57	57	50
Ferritin (ng/mL)	100	>4000	na	na	975	954	2531	820	155	>4000
Mean 1 yr Haemoglobin (g/dL)	12.2	9.5	7.9	na	9.2	8.5	9.5	10.2	10.3	9.2
Age commenced transfusions (years)	2	22	0.3	3	2	1	2	1	5	2
Units of blood in year before death/transplant	36	50	17	na	24	24	24	25	24	30
Total estimated units transfused	1584	2000	170	288	432	765	528	500	624	660
Age commenced chelation (years old)	6	42	No chelation	No chelation	6	10	6	4	7	10
Chelation in year before death/transplant	DFO, DFP	DFO	None	None	DFO	DFO	DFO	DFO	DFO	DFO, DFP
Hepatitis C	Yes	No	na	na	No	Yes	No	No	Yes	Yes
Diabetes	Yes	Yes	na	Yes	na	No	Yes	Yes	Yes	Yes
Splenectomy	Yes	No	Yes	Yes	Yes	Yes	Yes	Yes	Yes	Yes
Osteoporosis	Yes	No	na	Yes	No	No	No	No	No	Yes
Hypogonadism	Yes	No	Yes	Yes	No	No	No	Yes	No	na
Hypothyroid	Yes	No	Yes	Yes	No	No	No	Yes	No	na
Cardiac drugs	Diur Dig ACEI	ACEI	Dig	Dig	na	none	Dig Ami	CCB	Diur Dig	None
Other cardiac conditions	AF	AF	none	none	AF	none	AF	AF	none	none

*LV* left ventricle, *Tx* transplant, *TM* beta thalassemia major, *SA* sideroblastic anemia, *DFO* deferoxamine, *DFP* deferiprone, *AF* atrial fibrillation, *Diur* diuretic, *Dig* Digoxin, *ARB* Angiotensin receptor blocker, *CCB* Calcium channel blocker, *ACEI* Angiotensin converting enzyme inhibitor, *Ami* Amiodarone, *na* not available

### Tissue sampling and iron analysis

The left ventricle (LV) was cut into 5 short axis ventricular slices and ex-vivo CMR was performed on each slice, as described below. After imaging, each slice was divided into 6 sectors and 3 layers (epicardial, mesocardial and endocardial) making a total of 90 left ventricular samples per heart. Transmural samples were also taken of the right ventricle and both atria, and additional samples of the conduction tissue and valves. All samples were transported to a specialist lab for iron analysis in Perth, Australia. Each sample underwent lyophilization and acid digestion. Tissue iron concentration was measured using inductively coupled plasma atomic emission spectroscopy. Iron concentration values for the left ventricle samples showed only minor variation within each heart [19], and are therefore expressed as a mean whole heart iron concentration ± standard deviation.

### Histology

Whole blocks for histology were taken contiguous to the samples taken for iron analysis. Fifty blocks in each case was examined. The tissue blocks were fixed in formalin and dehydrated and embedded in paraffin wax. Sections were cut and stained with Picrosirius red (PSR) a stain which highlights collagen [20, 21]. Perl’s stain for iron detection [22, 23], and haematoxylin and eosin (H&E). Each slide was examined under a standard light microscope by two histopathologists for abnormalities, in particular the presence of fibrosis. Interstitial fibrosis was defined as increased interstitial and/or perivascular collagen without evidence of myocyte loss, or thin lines of collagen around individual myocytes. Replacement fibrosis was defined as myocyte replacement with collagen.

### Quantitative evaluation of fibrosis

After visual inspection, a representative digitized image was acquired from each slide of up to 1.8 mm^2^ (ventricular) or 0.3 mm^2^ (atrial). The images were analysed using dedicated computer software (Nikon NIS elements) which enabled a direct measurement of the myocardial tissue volume and PSR staining volume. The fraction of PSR staining within the myocardium was defined as the collagen volume fraction (CVF). Three studies published using this technique have shown a mean ± SD value of CVF for the left ventricle of 2.1 ± 0.45% [24–26], and for the right ventricle 2.5 ± 0.4% [23, 25]. Using the 2SD upper boundary this yields a normal value for CVF of <3% in the LV, and <3.3% in the RV. No normal quantitative values for the atria are available.

### Cardiovascular magnetic resonance

Cardiovascular magnetic resonance (CMR) T2* imaging is an established technique for assessing myocardial iron loading [19, 27–29]. Each of the 5 short-axis slices from each heart were scanned in-vitro at 37C to reproduce in-vivo body conditions. A 1.5 T scanner (Sonata, Siemens Medical Solutions, Erlangen, Germany) was used utilizing previously reported techniques [19, 30, 31]. In brief, a multi-echo T2* sequence (gradient echo) was used: Range of echo times TE from 3.1 to 39.1 ms; field of view 160x160mm; matrix 128x128; flip angle 20°; number of excitations 2; bandwidth 810Hz per pixel; TR 20 ms; slice thickness 5 mm. Data analysis was performed using CMR tools and its plug-in Thalassemia Tools (Cardiovascular Imaging Solutions, London UK).

## Results

### Heart iron deposition

**Fig. 1:**
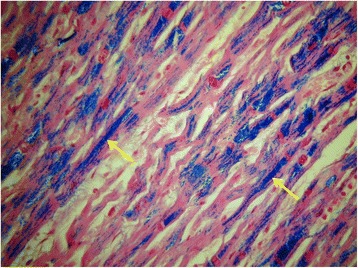
Heavily iron loaded myocardium (Perl’s stain). Nearly all myocytes showed homogenous positive purple/blue iron granules (arrows) within the cytoplasm (heart 4)

**Fig. 2:**
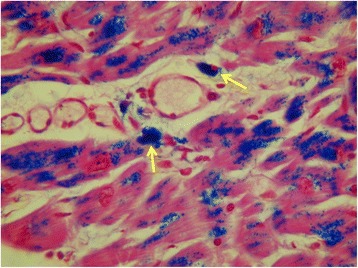
Homogenous granular staining with Perl’s stain of the cytoplasm of macrophages around capillaries (arrows) and myocytes (heart 3)

Nine hearts had severe iron loading with a mean ex-vivo LV T2* of 5.0 ± 2.0 ms and a mean LV iron concentration of 8.5 ± 7.0 mg/g dw (normal value <0.49 mg/g dw [32]) and a mean weight of 298 ± 63.7 g. There was diffuse granular iron deposition seen within all these 9 hearts. Nearly all myocytes showed homogenous positive purple/blue granules within the cytoplasm with Perl’s staining (Fig. 1). There was also homogenous positive granular staining of the cytoplasm of macrophages around blood vessels and within the interstitium of the myocardium (Fig. 2). A similar but reduced pattern of iron deposition was seen in the right ventricle and to a lesser degree in the myocytes of the atria. The normal fibrous subendocardium layer of the atria had little or no iron deposition, but positive staining was seen within scattered macrophages. The valves, nerves, ganglia, blood vessels, fat and connective tissue had little or no iron deposition apart from in scattered macrophages. There was 1 heart with no iron deposition that had a mean cardiac LV T2* of 44.4 ± 5.3 ms and a mean LV iron concentration of 0.38 ± 0.13 mg/g dw. This patient had died from a stroke with no heart failure. No iron deposition was seen with Perl’s stain.

### Picrosirius red staining for detecting fibrosis

**Fig. 3:**
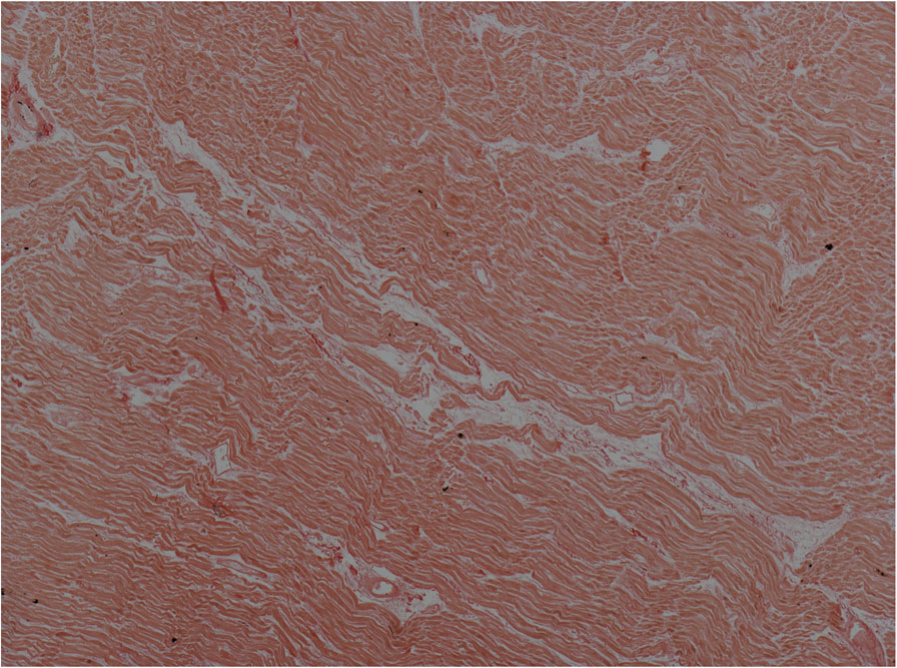
Picrosirius red staining of myocardium from a patient with severe cardiac siderosis (heart 3). No significant fibrosis is seen

The normal finding of PSR staining of collagen surrounding adventitia of blood vessels in the interstitium and the division of trabeculae was noted. Overall, the collagen volume fraction in all hearts was 2.28% ± 1.49%. No heart with cardiac siderosis had increased overall CVF (Fig. 3). There was occasional increased PSR staining at the insertion points of the RV into the LV in the anteroseptal and inferoseptal areas, which is a non-specific finding in the adult heart. The atrial PSR staining showed more prominent interstitial staining than that in the ventricles, which is also a normal finding [33]. The valves had prominent PSR staining of the zona fibrosa, which is a normal layer of collagen in all the valves. Detailed myocardial PSR quantification is shown in Tables 2 and 3.

**Table 2:** Percent collagen volume fraction (CVF) in left ventricle (normal <3%)

	Anterior wall	Lateral wall	Inferior wall	Septum
Heart 1	4.1%	8.9%	7.7%	2.9%
Heart 2	1.3%	1.8%	2.2%	1.2%
Heart 3	1.2%	1.7%	3.7%	1.3%
Heart 4	1.9%	1.9%	1.7%	1.5%
Heart 5	0.6%	0.5%	0.4%	0.7%
Heart 6	4.1%	2.8%	2.8%	2.4%
Heart 7	3.4%	3.1%	3.2%	2.5%
Heart 8	1.5%	2.1%	1.8%	1.4%
Heart 9	2.3%	2.2%	3.0%	1.7%
Heart10	0.7%	1.1%	1.2%	1.0%

**Table 3:** Percent collagen volume fraction (CVF) in right ventricle (normal <3.3%) and atria (no normal quantitative values for the atria are available)

	Anterior right ventricle	Posterior right ventricle	Right Atrium	Left Atrium
Heart 1	2.1%	1.4%	12.7%	26.5%
Heart 2	0.6%	1.6%	1.7%	11.2%
Heart 3	0.8%	1.8%	11.6%	8.5%
Heart 4	2.0%	2.6%	6.2%	23.9%
Heart 5	0.6%	1.2%	5.7%	4.8%
Heart 6	3.9%	3.5%	6.7%	17.0%
Heart 7	2.9%	2.8%	6.8%	5.2%
Heart 8	2.0%	1.5%	5.9%	6.7%
Heart 9	1.7%	3.0%	4.0%	14.7%
Heart 10	1.4%	1.2%	6.8%	3.2%

### Hearts with fibrosis

**Fig. 4:**
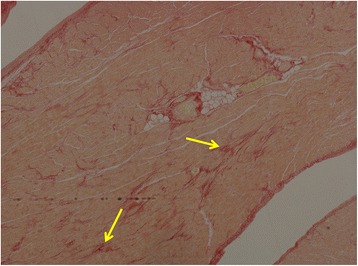
Mild patchy interstitial fibrosis identified with picrosirius red staining (arrows) was seen in a predominant subepicardial distribution in the left ventricle in heart 1 from a patient who died from a stroke, whose heart showed no cardiac iron

Fibrosis above the normal range was demonstrated in one heart by PSR staining (heart 1) with an overall CVF of 5.9 ± 2.8%, but this was considered too mild to cause heart failure. The donor had died from a stroke and had a long history of atrial fibrillation, but with no cardiac iron loading (LV T2* 44.4 ± 5.3 ms; mean LV iron concentration 0.38 ± 0.13 mg/g dw). The heart weighed 380 g with moderate left ventricular hypertrophy. Minimal fibrosis was noted in the mid left ventricular inferoseptal wall and basal anteroseptal wall. Mild patchy interstitial fibrosis was also seen which was predominantly subepicardial (Fig. 4). Heart 3 also had minor fibrosis which in some scattered areas (subepicardial anterior wall) was above the normal range, but the overall mean left ventricular CVF was normal at 2.0 ± 1.1%. Severe iron loading was present, with mean LV T2* of 3.7 ± 0.6 ms, mean LV iron concentration of 9.5 ± 1.9 mg/g dw and severe granular iron deposition on Perl’s stain. The heart was small with a weight of 236 g. The donor had severe heart failure as the cause of death. The level of fibrosis was considered too minimal to cause heart failure. There was no evidence of white cell infiltration to suggest myocarditis.

## Discussion

This study failed to show replacement cardiac fibrosis as a cause of death or transplantation related to heart failure in patients with transfusion dependent anaemia, including most importantly beta-thalassaemia major patients. These findings are in marked contrast to the major historical pathological series in transfusion dependent patients. In 1964 [4], post-mortem data published by Engle on 41 chronic anemia patients (39 with thalassemia major, 2 with aplastic anemia), showed 26 (63%) had heart failure with a further 6 (15%) having cardiac enlargement and an abnormal ECG. These data clearly indicated that the heart was the target lethal organ in beta-thalassaemia major. Engle noted that focal myocyte degeneration and fibrosis was extensive, and was an “adequate explanation for the progressive cardiac enlargement and heart failure”. In 1971 [5], Buja reported post-mortem findings on 19 patients with cardiac iron deposits (8 with aplastic anemia, 4 with chronic myelocytic leukemia, 3 with idiopathic hemochromatosis, 2 with chronic lymphocytic leukemia, 1 with acute lymphocytic leukemia, 1 with sickle cell anemia). Heart failure occurred in 14 (67%) patients. The left ventricle was affected by extensive myocardial replacement fibrosis in 6 (29%) patients and by focal interstitial fibrosis in 4 (21%) patients. The papillary muscles were affected by focal interstitial fibrosis in a further 6 (29%) patients. In only 3 patients (14%), was the heart not affected by fibrosis. Buja listed a further 21 reports dating from 1933 to 1967 as showing myocardial fibrosis in patients with cardiac iron loading [5].

The cause of cardiac fibrosis in iron overload conditions is not fully explained. The most important effect appears to be that myocytes can suppress proliferation of cardiac fibroblasts by cumulative effects on late G1 events leading to DNA synthesis, and these effects are diminished with myocyte iron accumulation, which encourages cardiac fibrosis [34, 35]. However, a less supporting study of mRNA in ex-vivo cardiac myocytes showed iron level dependent reductions in expression of transforming growth factor-β1 (TGF-B1), biglycan, and collagen type I, which was accompanied by a reduction in TGF-B1 bioactivity, which does not obviously support iron-driven cardiac fibrogenesis [36]. Animal models support a link between cardiac iron and cardiac fibrosis. Cardiac fibrosis was prominent in double knock-out mice for beta 2 microglobulin (B2m, deficiency of which causes increased gut uptake of iron through impairment of the HFE-B2m complex) and recombinase activator gene 1 (Rag1, deficiency of which causes absence of B and T lymphocytes) which was not seen in B2m and Rag1 single-knockout mice or control mice of the same age, implying that lymphocytes play a role in cardiac fibrosis which is additive to cardiac iron loading alone [37]. Other iron loading animal models also show cardiac fibrosis, although this was not prominent [38, 39].

The explanation for the apparent change in prevalence of replacement cardiac fibrosis over 40 years can only be subject to speculation. One obvious possibility is the introduction of the iron chelator deferoxamine, which came into widespread clinical use in the 1970’s. Although not explicitly stated, the patients in Engle and Buja’s papers would not have received such treatment based on the period of the patient post-mortems (1950–1963, and 1953–1969 respectively). This suggests that deferoxamine might impair the development cardiac fibrosis that occurs with myocardial siderosis. This is plausible as deferoxamine is known to stabilise liver fibrosis in association with reduced liver iron loading [40]. There is also experimental evidence which supports this position. Control of cardiac iron may in itself prevent cardiac fibrosis by suppressing fibroblast proliferation [34, 35] but other direct supportive evidence for a protective effect of deferoxamine comes from studies of angiotensin II in normal and iron loaded rats in which the development of cardiac fibrosis could be prevented by deferoxamine [41]. Since angiotensin II causes cardiac fibrosis and is increased in cardiac failure, this effect could be clinically significant. However, this explanation is not completely sufficient because patients 3 and 4 in our series, did not receive chelation but did not have significant myocardial fibrosis.

Although the use of deferoxamine seems the most likely factor distinguishing the historical from our modern cardiac findings, other possibilities exist. Another change in treatment of thalassemia major patients is the use of increased numbers of blood transfusions per year since the 1970’s, which suppresses ineffective erythropoiesis and bony abnormality. A typical modern regime for an adult includes the transfusion of up to 50 units of blood per year (0.4 mg/kg/day transfusional iron burden) [42]. Engle documented a transfusion rate on average of approximately 13.4 units/year in 26 patients who developed heart failure [4], consistent with a >3 fold increase in transfusion rate for modern patients. Cardiac fibrosis could therefore have been stimulated by anemia related myocardial hypoxia in historical patient cohorts, particularly in the setting of left ventricular hypertrophy and dilatation [43]. Increased transfusions with improved tissue oxygenation might have played a role therefore in reducing myocardial fibrosis. The change in cardiac fibrosis might also be related to an apparent reduction in recurrent pericarditis seen in the last 40 years. Engle reported 19 of 41 (46%) patients had 33 recognised episodes of pericarditis [4]. Pericarditis was not a focus of Buja’s paper [5]. Pericarditis is recognized in thalassemia major in the modern era, but at a far lower incidence of <5% [44]. It is likely that pericarditis is now less frequent because of the widespread use of iron chelation therapy, but the genesis of the pericarditis in iron overload is not well understood and other factors might be important. Myocarditis is also recognised as a cause of myocardial fibrosis [45], and has been documented from a modern series as occurring in 4.5% of beta-thalassemia major patients [46]. Myocarditis was not documented in the historical series and direct comparisons with the modern findings are therefore not possible. However, the fact that the detailed historical papers did not report myocardial inflammation, would not suggest that it was prominent or common, and therefore a reduction in prevalence seems unlikely. It should be noted that in the modern environment, approximately 2% of cases of heart failure in thalassemia major have low levels of cardiac iron [47], and these are thought to be caused by myocarditis [48], in which cardiac fibrosis may play a significant role [45], which is in addition to the myocardial infection and inflammation. Another possibility is that a historical factor in blood transfusion practice was associated with cardiac fibrosis in the past that has now decreased. The reduced transmission of cardiotrophic viruses might be a possibility, of which one candidate virus is hepatitis C, which has been implicated in myocarditis and cardiac fibrosis [49–51]. Reports of infrequent cardiac fibrosis in beta-thalassemia major using the non-invasive in-vivo technique of late gadolinium enhancement (LGE) CMR showed infrequent minor fibrosis (24%, 2% and 15.8% of patients) in patients without heart failure [52–54], but the extent of fibrosis was limited (3.9%, 0.4%, 1.3%) [52–54]. In comparison to other disease settings it is not clear whether this minor fibrosis would be sufficient to cause significant LV dysfunction, where on average each 1% of infarcted myocardium assessed by LGE leads to only a modest 0.67% reduction in ejection fraction [55]. However, the difference in prevalence of minor cardiac fibrosis between centers might be explained by the different prevalence of hepatitis C infection [52, 53]. Despite this possibility however, extensive cardiac fibrosis causing heart failure is not seen in hepatitis C infection, and it is unlikely that changes in transfusional infections can explain the change over time in replacement cardiac fibrosis.

Further possible factors that might influence the difference in cardiac fibrosis over time are: 1) the occurrence of diabetes in thalassaemia major, which has association with the presence of cardiac fibrosis and development of heart failure [56]. However, seven of our 10 patients had confirmed diabetes without significant cardiac fibrosis. 2) the use of inhibitors of the renin-angiotensin-aldosterone system such as angiotensin converting enzyme inhibitors, angiotensin receptor blockers (ARB) and mineralocorticoid receptor antagonists (MRA) which are known potent inhibitors of myocardial fibrosis [57–60]. Only 2 of our patients were recorded as being on such treatment, although due to the retrospective nature of data collection in this series, the accuracy of drug treatments may not be ideal. 3) Finally, in older transfusion dependent patients, the pattern of heart failure and the relationship to cardiac fibrosis may differ in comparison with younger patients, with development of heart failure with preserved ejection fraction [61]. This cardiac pathology is not completely understood but restrictive physiology is involved.

### Limitations

The absence of in-vivo CMR does not allow the comparison of late gadolinium enhancement with the histological findings. The technique of T1 mapping was likewise not possible in-vivo in this population [62]. This is inevitable given the international source of the post-mortem hearts. This was a small study in comparison with historical reports, which reflects the general trend towards reduction of patients undergoing post-mortem.

## Conclusion

Severe myocardial siderosis causes a toxic dilated cardiomyopathy which can be reversed if aggressive chelation is commenced early. In the current study, the direct histological examination of hearts from patients with terminal heart failure has shown no significant replacement myocardial fibrosis. This suggests that the cause of left ventricular impairment seen in cardiac siderosis is a direct result of myocardial iron toxicity and not due to fibrosis. The discrepancy of these findings in comparison with historical reports of extensive fibrosis in cardiac siderosis is most probably explained by the use of iron chelation treatment, although other factors may play a role including increased transfusions.

## Abbreviations

CMR: Cardiovascular magnetic resonance
CVF: Collagen volume fraction
H&E: Haematoxylin and eosin
LV: Left ventricle
PSR: Picrosirius red
TGF-B1: Transforming growth factor-β1

## References

[1] Weatherall DJ. Anaemia as a World Health Problem. Oxford Textbook of Medicine. Oxford: Oxford University Press; 1996. 3463–82.

[2] Modell B, Khan M, Darlison M (2000). Survival in beta-thalassaemia major in the UK: data from the UK Thalassaemia Register. Lancet.

[3] Modell B, Khan M, Darlison M, Westwood MA, Ingram D, Pennell DJ (2008). Improved survival of thalassaemia major in the UK and relation to T2* cardiovascular magnetic resonance. J Cardiovasc Magn Reson.

[4] Engle MA, Erlandson M, Smith CH (1964). Late cardiac complications of chronic, severe, refractory anemia with hemochromatosis. Circulation.

[5] Buja LM, Roberts WC (1971). Iron in the heart. Etiology and clinical significance. Am J Med.

[6] Kirk P, Roughton M, Porter JB, Walker JM, Tanner MA, Patel J, Wu D, Taylor J, Westwood MA, Anderson LJ, Pennell DJ (2009). Cardiac T2* magnetic resonance for prediction of cardiac complications in thalassemia major. Circulation.

[7] Anderson LJ, Westwood MA, Holden S, Davis B, Prescott E, Wonke B, Porter JB, Walker JM, Pennell DJ (2004). Myocardial iron clearance during reversal of siderotic cardiomyopathy with intravenous desferrioxamine: a prospective study using T2* cardiovascular magnetic resonance. Br J Haematol.

[8] Pennell DJ, Berdoukas V, Karagiorga M, Ladis V, Piga A, Aessopos A, Gotsis ED, Tanner MA, Smith GC, Westwood MA, Wonke B, Galanello R (2006). Randomized controlled trial of deferiprone or deferoxamine in beta-thalassemia major patients with asymptomatic myocardial siderosis. Blood.

[9] Tanner MA, Galanello R, Dessi C, Smith GC, Westwood MA, Agus A, Roughton M, Assomull R, Nair SV, Walker JM, Pennell DJ (2007). A randomized, placebo-controlled, double-blind trial of the effect of combined therapy with deferoxamine and deferiprone on myocardial iron in thalassemia major using cardiovascular magnetic resonance. Circulation.

[10] Tanner MA, Galanello R, Dessi C, Smith GC, Westwood MA, Agus A, Pibiri M, Nair SV, Walker JM, Pennell DJ (2008). Combined chelation therapy in thalassemia major for the treatment of severe myocardial siderosis with left ventricular dysfunction. J Cardiovasc Magn Reson.

[11] Moon JC, Reed E, Sheppard MA, Elkington AG, Ho SY, Burke M, Petrou M, Pennell DJ (2004). The histological basis of late gadolinium enhancement cardiovascular magnetic resonance in hypertrophic cardiomyopathy. J Am Coll Cardiol.

[12] McCrohon JA, Moon JC, Prasad SK, McKenna WJ, Lorenz CH, Coats AJ, Pennell DJ (2003). Differentiation of heart failure related to dilated cardiomyopathy and coronary artery disease using gadolinium-enhanced cardiovascular magnetic resonance. Circulation.

[13] Tandri H, Saranathan M, Rodriguez ER, Martinez C, Bomma C, Nasir K, Rosen B, Lima JA, Calkins H, Bluemke DA (2005). Noninvasive detection of myocardial fibrosis in arrhythmogenic right ventricular cardiomyopathy using delayed-enhancement magnetic resonance imaging. J Am Coll Cardiol.

[14] Sen-Chowdhry S, Syrris P, Prasad SK, Hughes SE, Merrifield R, Ward D, Pennell DJ, McKenna WJ (2008). Left-dominant arrhythmogenic cardiomyopathy: an under-recognized clinical entity. J Am Coll Cardiol.

[15] Assomull RG, Prasad SK, Lyne J, Smith G, Burman ED, Khan M, Sheppard MN, Poole-Wilson PA, Pennell DJ (2006). Cardiovascular magnetic resonance, fibrosis, and prognosis in dilated cardiomyopathy. J Am Coll Cardiol.

[16] Moon JC, McKenna WJ, McCrohon JA, Elliott PM, Smith GC, Pennell DJ (2003). Toward clinical risk assessment in hypertrophic cardiomyopathy with gadolinium cardiovascular magnetic resonance. J Am Coll Cardiol.

[17] O’Hanlon R, Grasso A, Roughton M, Moon JC, Clark S, Wage R, Webb J, Kulkarni M, Dawson D, Sulaibeekh L, Chandrasekaran B, Bucciarelli-Ducci C, Pasquale F, Cowie MR, McKennaWJ SMN, Elliott PM, Pennell DJ, Prasad SK (2010). Prognostic significance of myocardial fibrosis in hypertrophic cardiomyopathy. J Am Coll Cardiol.

[18] Bruder O, Wagner A, Jensen CJ, Schneider S, Ong P, Kispert EM, Nassenstein K, Schlosser T, Sabin GV, Sechtem U, Mahrholdt H (2010). Myocardial scar visualized by cardiovascular magnetic resonance imaging predicts major adverse events in patients with hypertrophic cardiomyopathy. J Am Coll Cardiol.

[19] Carpenter JP, He T, Kirk P, Roughton M, Anderson LJ, de Noronha SV, Sheppard MN, Porter JB, Walker JM, Wood JC, Galanello R, Forni G, Catani G, Matta G, Fucharoen S, Fleming A, Black G, Firmin DN, St Pierre TG, Pennell DJ, House MJ (2011). On T2* magnetic resonance and cardiac iron. Circulation.

[20] Weatherford TW (1972). Staining of collagenous and non-collagenous structures with picrosirius red F3BA. Ala J Med Sci.

[21] Whittaker P, Kloner RA, Boughner DR, Pickering JG (1994). Quantitative assessment of myocardial collagen with p picrosirius red staining and circularly polarized light. Basic Res Cardiol.

[22] Dumont JN, Cone MV (1970). Ultrastructural localization of iron by Perls’ or by Turnbull’s method applied to tissue prior to embedding. Stain Technol.

[23] Tanaka Y, Berschauer JA (1969). Application of the Perls method for iron staining to sections embedded in epoxy resin. Stain Technol.

[24] Volders PG, Willems IE, Cleutjens JP, Arends JW, Havenith MG, Daemen MJ (1993). Interstitial collagen is increased in the non-infarcted human myocardium after myocardial infarction. J Mol Cell Cardiol.

[25] Tanaka M, Fujiwara H, Onodera T, Wu DJ, Hamashima Y, Kawai C (1986). Quantitative analysis of myocardial fibrosis in normals, hypertensive hearts, and hypertrophic cardiomyopathy. Br Heart J.

[26] John BT, Tamarappoo BK, Titus JL, Edwards WD, Shen WK, Chugh SS (2004). Global remodeling of the ventricular interstitium in idiopathic myocardial fibrosis and sudden cardiac death. Heart Rhythm.

[27] Anderson LJ, Holden S, Davis B, Prescott E, Charrier CC, Bunce NH (2001). Cardiovascular T2-star (T2*) magnetic resonance for the early diagnosis of myocardial iron overload. Eur Heart J.

[28] Westwood MA, Firmin DN, Gildo M, Renzo G, Stathis G, Markissia K (2005). Intercentre reproducibility of magnetic resonance T2* measurements of myocardial iron in thalassaemia. Int J Cardiovasc Imaging.

[29] Tanner MA, He T, Westwood MA, Firmin DN, Pennell DJ (2006). Multi-center validation of the transferability of the magnetic resonance T2* technique for the quantification of tissue iron. Haematologica.

[30] Westwood M, Anderson LJ, Firmin DN, Gatehouse PD, Charrier CC, Wonke B, Pennell DJ (2003). A single breath-hold multiecho T2* cardiovascular magnetic resonance technique for diagnosis of myocardial iron overload. J Magn Reson Imaging.

[31] He T, Gatehouse PD, Smith GC, Mohiaddin RH, Pennell DJ, Firmin DN (2008). Myocardial T2* measurements in iron-overloaded thalassemia: An ex vivo study to investigate optimal methods of quantification. Magn Reson Med.

[32] Collins W, Taylor WH (1987). Determination of iron in cardiac and liver tissues by plasma emission spectroscopy. Ann Clin Biochem.

[33] Milliez P, Deangelis N, Rucker-Martin C, Leenhardt A, Vicaut E, Robidel E, Beaufils P, Delcayre C, Hatem SN (2005). Spironolactone reduces fibrosis of dilated atria during heart failure in rats with myocardial infarction. Eur Heart J.

[34] Liu Y, Templeton DM (2001). The effects of cardiac myocytes on interstitial fibroblasts in toxic iron overload. Cardiovasc Toxicol.

[35] Liu Y, Templeton DM (2006). Iron-loaded cardiac myocytes stimulate cardiac myofibroblast DNA synthesis. Mol Cell Biochem.

[36] Parkes JG, Ying Liu Y, Sirna JB, Templeton DM (2000). Changes in gene expression with iron loading and chelation in cardiac myocytes and non-myocytic fibroblasts. J Mol Cell Cardiol.

[37] Santos MM, de Sousa M, Rademakers LH, Clevers H, Marx JJ, Schilham MW (2000). Iron overload and heart fibrosis in mice deficient for both beta2-microglobulin and Rag1. Am J Pathol.

[38] Carthew P, Dorman BM, Edwards RE, Francis JE, Smith AG (1993). A unique rodent model for both the cardiotoxic and hepatotoxic effects of prolonged iron overload. Lab Invest.

[39] Wood JC, Otto-Duessel M, Aguilar M, Nick H, Nelson MD, Coates TD, Pollack H, Moats R (2005). Cardiac iron determines cardiac T2*, T2, and T1 in the gerbil model of iron cardiomyopathy. Circulation.

[40] Barry M, Flynn DM, Letsky EA, Risdon RA (1974). Long-term chelation therapy in thalassaemia major: effect on liver iron concentration, liver histology, and clinical progress. Br Med J.

[41] Ishizaka N, Saito K, Mitani H, Yamazaki I, Sata M, Usui SI, Mori I, Ohno M, Nagai R (2002). Iron overload augments angiotensin II–induced cardiac fibrosis and promotes neointima formation. Circulation.

[42] Cohen AR, Glimm E, Porter JB (2008). Effect of transfusional iron intake on response to chelation therapy in beta-thalassemia major. Blood.

[43] Sonakul D, Thakerngpol K, Pacharee P (1988). Cardiac pathology in 76 thalassemic patients. Birth Defects Orig Artic Ser.

[44] Aessopos A, Farmakis D, Hatziliami A, Fragodimitri C, Karabatsos F, Joussef J, Mitilineou E, Diamanti-Kandaraki E, Meletis J, Karagiorga M (2004). Cardiac status in well-treated patients with thalassemia major. Eur J Haematol.

[45] Mahrholdt H, Wagner A, Deluigi CC, Kispert E, Hager S, Meinhardt G, Vogelsberg H, Fritz P, Dippon J, Bock CT, Klingel K, Kandolf R, Sechtem U (2006). Presentation, patterns of myocardial damage, and clinical course of viral myocarditis. Circulation.

[46] Kremastinos DT, Tiniakos G, Theodorakis GN, Katritsis DG, Toutouzas PK (1995). Myocarditis in beta-thalassemia major. A cause of heart failure. Circulation.

[47] Carpenter JP, Roughton M, Pennell DJ (2012). International survey of T2* cardiovascular magnetic resonance in thalassemia. Haematologica.

[48] Roghi A, Dellegrottaglie S, Pedrotti P, Pedretti S, Cassinerio E, Cappellini MD. Unexpected myocarditis in thalassaemia major patient screened for iron load cardiomyopathy. BMJ Case Rep. 2009;2009. doi: 10.1136/bcr.08.2008.0811. 10.1136/bcr.08.2008.0811 PMC3027640 21686619

[49] Omura T, Yoshiyama M, Hayashi T, Nishiguchi S, Kaito M, Horiike S, Fukuda K, Inamoto S, Kitaura Y, Nakamura Y, Teragaki M, Tokuhisa T, Iwao H, Takeuchi K, Yoshikawa J (2005). Core protein of hepatitis C virus induces cardiomyopathy. Circ Res.

[50] Matsumori A, Shimada T, Chapman NM, Tracy SM, Mason JW (2006). Myocarditis and heart failure associated with hepatitis C virus infection. J Card Fail.

[51] Sanchez MJ, Bergasa NV (2008). Hepatitis C associated cardiomyopathy: potential pathogenic mechanisms and clinical implications. Med Sci Monit.

[52] Pepe A, Positano V, Capra M, Maggio A, Pinto CL, Spasiano A, Forni G, Derchi G, Favilli B, Rossi G, Cracolici E, Midiri M, Lombardi M (2009). Myocardial scarring by delayed enhancement cardiovascular magnetic resonance in thalassaemia major. Heart.

[53] Kirk P, Carpenter JP, Tanner MA, Pennell DJ (2011). Low prevalence of fibrosis in thalassemia major assessed by late gadolinium enhancement cardiovascular magnetic resonance. J Cardiovasc Magn Reson.

[54] Casale M, Meloni A, Filosa A, Cuccia L, Caruso V, Palazzi G, Gamberini MR, Pitrolo L, Putti MC, D’Ascola DG, Casini T, Quarta A, Maggio A, Neri MG, Positano V, Salvatori C, Toia P, Valeri G, Midiri M, Pepe A (2015). Multiparametric cardiac magnetic resonance survey in children with thalassemia major: A multicenter study. Circ Cardiovasc Imaging.

[55] Shriki JE, Surti K, Farvid A, Shinbane JS, Colletti PM (2009). Quantitative evaluation of the amount of delayed myocardial enhancement as a predictor of systolic dysfunction. Open Cardiovasc Med J.

[56] Pepe A, Meloni A, Rossi G, Caruso V, Cuccia L, Spasiano A, Gerardi C, Zuccarelli A, D’Ascola DG, Grimaldi S, Santodirocco M, Campisi S, Lai ME, Piraino B, Chiodi E, Ascioti C, Gulino L, Positano V, Lombardi M, Gamberini MR (2013). Cardiac complications and diabetes in thalassaemia major: a large historical multicentre study. Br J Haematol.

[57] González A, López B, Díez J (2004). Fibrosis in hypertensive heart disease: role of the renin-angiotensin-aldosterone system. Med Clin North Am.

[58] Roubille F, Busseuil D, Merlet N, Kritikou EA, Rhéaume E, Tardif JC (2014). Investigational drugs targeting cardiac fibrosis. Expert Rev Cardiovasc Ther.

[59] Kawano H, Toda G, Nakamizo R, Koide Y, Seto S, Yano K (2005). Valsartan decreases type I collagen synthesis in patients with hypertrophic cardiomyopathy. Circ J.

[60] Brilla CG, Funck RC, Rupp H (2000). Lisinopril-mediated regression of myocardial fibrosis in patients with hypertensive heart disease. Circulation.

[61] Bakeer N, James J, Roy S, Wansapura J, Shanmukhappa SK, Lorenz JN, Osinska H, Backer K, Huby AC, Shrestha A, Niss O, Fleck R, Quinn CT, Taylor MD, Purevjav E, Aronow BJ, Towbin JA, Malik P (2016). Sickle cell anemia mice develop a unique cardiomyopathy with restrictive physiology. Proc Natl Acad Sci U S A.

[62] Hanneman K, Nguyen ET, Thavendiranathan P, Ward R, Greiser A, Jolly MP, Butany J, Yang IY, Sussman MS, Wintersperger BJ (2016). Quantification of myocardial extracellular volume fraction with cardiac MR imaging in thalassemia major. Radiology.

